# Interleukin-37 mediates the antitumor activity in colon cancer through β-catenin suppression

**DOI:** 10.18632/oncotarget.17093

**Published:** 2017-04-13

**Authors:** Xiaofei Yan, Jian Zhao, Rui Zhang

**Affiliations:** ^1^ Department of Colorectal Surgery, Liaoning Cancer Hospital & Institute, Cancer Hospital of China Medical University, Shenyang 110042, Liaoning Province, P.R. China

**Keywords:** interleukin-37, colon cancer, β-catenin, IL-37

## Abstract

The occurrence and development of colon cancer is closely related to inflammation. Thus, we conducted the present retrospective study to investigate the effects of IL-37 (Interleukin 37), a newly identified anti-inflammatory factor, on colon cancer development. We first evaluated the IL-37 expression in 186 pairs of colon cancer samples and their adjacent normal mucosa by real-time PCR, ELISA (Enzyme-linked immunoassay) and tissue microarrays. Then the role of IL-37 on patient survival rates, colon cancer progression and their sensitivity to chemotherapy drugs were assessed. IL-37 was barely expressed in the colon cancer tissue but highly expressed in the adjacent normal tissue. The down-regulation of IL-37 was significantly correlated with the results of American Joint Committee on Cancer stage, nodal involvement, invasion depth, distant metastasis, differentiation and it was also shown to be an independent prognostic indicator of disease-free survival and overall survival for patients with colon cancer. Overexpression of IL-37 in colon cancer cell suppressed cell migration, invasion, proliferation, colony formation and cancer stem cells through suppressing β-catenin. IL-37 inhibited colon tumor formation in the mice model and sensitize the cancer cell to chemotherapy drugs. Our results showed that IL-37 plays an inhibitory role in colon cancer development and function as a novel prognostic indicator and a potential therapeutic target.

## INTRODUCTION

Colon cancer is one of the most prevalent malignancies worldwide [1]. It is estimated that over half of the colon cancer patients had developed distant metastasis [2]. Big progress has been made in cancer detection and treatment during the past decades; however, the 5-year survival rate for colon cancer at distant stage is extremely low. Thus, identifying more available biomarkers, determining the mechanisms involved in colon cancer development and novel therapeutic targets development are necessary.

Colon cancer development is a multistage process, which originates from normal mucosa, and then adenomatous polyps (adenoma), carcinoma *in situ*, and ultimately to invasive and metastatic carcinoma, resulting from genetic mutation or chronic inflammation [3]. Previous studies have demonstrated that multiple mechanisms are responsible for the colon cancer development. Recently, growing evidences indicate that immune mechanisms are important in colon cancer progression [4]. Dysregulated inflammatory response is related to an increased risk of chronic disease and cancers. And pro-inflammatory cytokines are related to tumor development, including proliferation, metastasis, apoptosis and angiogenesis [5]. IL-37 (Interleukin-37) has shown anti-inflammatory and immune suppression effects [6–8]. Recently, it has been demonstrated that IL-37 suppresses tumor progression, including cervical cancer [9], fibrosarcoma [10], hepatocellular carcinoma [11], lung cancer [12, 13], renal cell carcinoma[14] and breast cancer [15]. The underlying mechanisms were proposed as CD57^+^ NK recruitment [11], IL-6/STAT3 signaling suppression [9, 14], angiogenesis and epithelial-mesenchymal transition inhibition [12, 13].

However, whether IL-37 also shows anti-tumor effects in colon cancer remains unknown. Therefore, we investigated the role of IL-37 in colon cancer in the current study.

## RESULTS

### Decreased IL-37 expression in colon cancer biopsies

**Figure 1:**
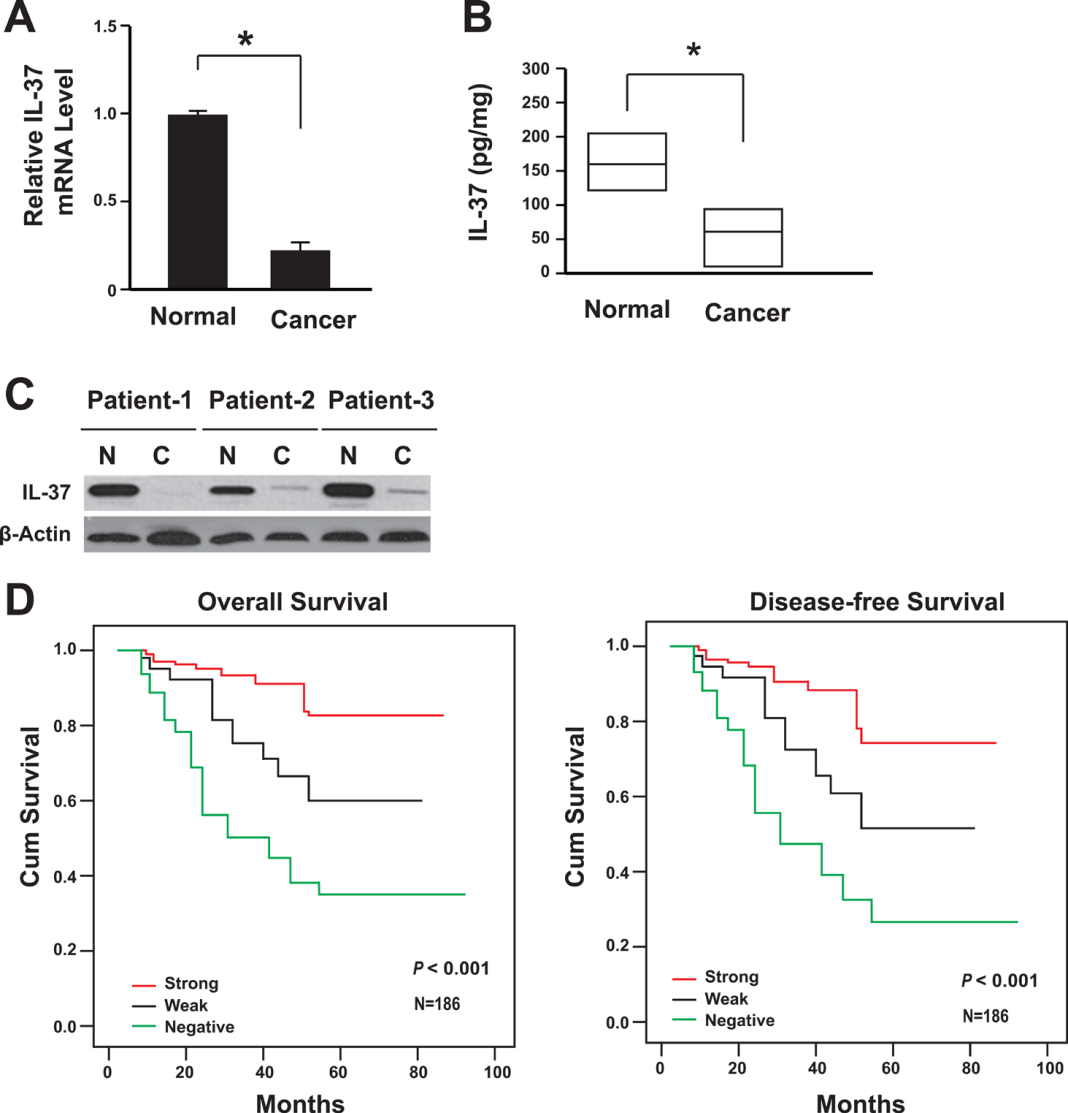
Lack of IL-37 expression correlated with the poor prognosis of colon cancer (**A**) IL-37 mRNA levels in cancer tissues and adjacent normal tissues were determined by real-time PCR (*n* = 186). *P < 0.05. (**B**) IL-37 protein levels in cancer tissues (95% CI: 11.5–109.6 pg/mg) and adjacent normal tissues (95% CI: 119.1–216.9 pg/mg) were determined by ELISA (*n* = 186). Result is depicted as box plots; middle line indicates median; bottom of box, 25th percentile; and top of box, 75th percentile. *P < 0.05. (**C**) Representative figure for IL-37 protein levels in cancer tissues and adjacent normal tissues were determined by western blot. N: adjacent normal tissues; C: cancer tissues. (**D**) Kaplan-Meier survival curve of patients with negative, weak or strong expression of IL-37.

A total of 186 cases with colon cancer were followed. All these patients had received no pre-operation chemotherapy. They were given the same radical operation and underwent the same adjuvant chemotherapy after the surgery. The IL-37 expression was firstly analyzed in 186 colon specimens. The data showed that the IL-37 expression decreased at both mRNA and protein levels in colon cancer tissues comparing with its paired adjacent non-cancerous tissues (Figure 1A and 1B), and this was further validated with western blot (Figure 1C).

Immunohistochemical analysis on a tissue array showed that the lack of IL-37 expression was associated with lymph nodes metastasis significantly (Table 1, Supplementary Figure 1A and 1B). The lack of IL-37 expression also showed association with the AJCC stage (*P* = 0.011), differentiation (*P* = 0.001), nodal involvement (*P* = 0.009), invasion (*P* = 0.018) and metastasis (*P* = 0.002).

**Table 1:** Association between clinicopathological features and IL-37 protein expression

	*N*	IL-37 expression			*P* value
		Strong (*n* = 62, %)	Weak (*n* = 54, %)	Negative (*n* = 70, %)	
Age, years					0.393
< 65	72	34.0	37.0	45.0	
≥ 65	114	66.0	63.0	55.0	
Gender					0.521
Male	80	48.4	40.7	39.1	
Female	106	51.6	59.3	60.9	
Location					0.651
Right	74	41.9	35.2	42.0	
Transverse	18	11.2	11.1	7.2	
Left	19	4.8	13.0	13.1	
Sigmoid colon	75	42.1	40.7	37.7	
T stage					0.018*
T1	8	3.2	5.5	3.0	
T2	21	16.1	13.0	5.7	
T3	72	46.9	42.5	29.1	
T4	85	33.8	39.0	62.2	
N stage					0.009*
N0	96	67.7	48.1	40.7	
N1	58	25.7	33.3	34.7	
N2	32	6.6	18.6	24.6	
M stage					0.002*
M0	168	96.8	96.3	81.3	
M1	18	3.2	3.7	18.7	
AJCC stage					0.011*
I	22	17.7	11.0	7.2	
II	71	46.8	37.0	31.9	
III	75	32.2	48.0	42.0	
IV	18	3.3	4.0	18.9	
Differentiation					0.001*
High	90	66.0	50.0	32.0	
Moderate	68	27.4	36.0	41.0	
Low	28	6.6	14.0	27.0	
Vascular invasion					0.422
Yes	173	96.8	92.6	91.4	
No	13	3.2	7.4	8.6	

*P < 0.05 indicates a significant association among the variables.

During the follow-up for all patients, 62 patients had died and 75 experienced recurrence. Disease-free survival (DFS) and overall survival (OS) was conducted to assess the predictive role of IL-35 for distant metastasis. Both DFS and OS were significantly higher in IL-37 positive groups (both weak and strong expression of IL-37) than the negative group (Figure 1D). The IL-37 negative group subsequently developed more recurrence or metastasis than IL-37 positive groups (*P <* 0.01).

Univariate analysis showed that patients with IL-37 negative group had a significantly reduced OS and DFS than the IL-37 positive groups (Table 2). Furthermore, the lack of IL-37 expression was showed as an independent prognostic marker for colon tumor recurrence (Table 3).

**Table 2:** Univariate Cox proportional hazards model for disease-free survival (DFS) and overall survival (OS)

	DFS			OS		
	HR	95% CI	*P* value	HR	95% CI	*P* value
Age, years						
< 65	—			—		
≥ 65	1.009	0.659–1.841	0.714	0.934	0.530–1.644	0.812
Gender						
Male	—			—		
Female	1.013	0.617–1.662	0.961	1.546	0.860–2.776	0.145
Tumor location						
Right	—			—		
Transverse	0.816	0.309–2.155	0.681	0.789	0.267–2.334	0.669
Left	1.392	0.619–3.130	0.423	1.291	0.512–3.252	0.589
Sigmoid colon	1.251	0.718–2.176	0.431	1.145	0.614–2.135	0.671
T stage						
T1	0.494	0.119–2.049	0.331	0.988	0.299–3.259	0.984
T2	0.203	0.063–0.660	0.006*	0.394	0.138–1.130	0.083
T3	0.484	0.284–0.827	0.004*	0.521	0.280–0.966	0.019*
T4	—			—		
N stage						
N0	—			—		
N1	5.887	3.025–11.456	< 0.001*	4.157	2.009–8.602	< 0.001*
N2	15.914	7.781–32.545	< 0.001*	13.193	6.149–28.307	< 0.001*
AJCC stage						
I	—			—		
II	1.108	0.305–4.027	0.877	0.667	0.201–2.215	0.508
III	6.823	2.097–22.199	0.001*	3.401	1.184–9.771	0.023*
IV	49.185	12.615–191.764	< 0.001*	40.074	11.257–142.668	< 0.001*
Differentiation						
High	—			—		
Moderate	1.315	0.750–2.306	0.341	1.458	0.764–2.780	0.253
Low	3.577	1.885–6.786	< 0.001*	4.358	2.140–8.872	< 0.001*
Vascular invasion						
Yes	4.901	2.469–9.721	< 0.001*	4.638	2.152–9.997	< 0.001*
No	—			—		
IL-37 expression						
Positive	—			—		
Weak	2.598	1.194–5.653	0.011*	2.117	0.862–5.196	0.102
Negative	6.118	3.004–12.462	< 0.001*	6.348	2.875–14.014	< 0.001*

*P < 0.05 indicates a significant association among the variables.

**Table 3:** Multivariate Cox proportional hazards model for DFS and OS

	DFS			OS		
	HR	95% CI	*P* value	HR	95% CI	*P* value
IL-37 expression	2.796	1.919–4.161	< 0.001*	2.659	1.711–4.223	< 0.001*
T stage	1.701	1.129–2.541	0.008*	3.981	1.854–9.173	< 0.001*
N stage	3.698	2.049–6.701	< 0.001*	3.321	1.813–6.203	< 0.001*
M stage	4.402	1.299–14.551	0.011*	8.001	2.403–26.815	< 0.001*

*P < 0.05 indicates a significant association among the variables.

Taken together, the data showed that the reduced IL-37 expression might contribute to colon cancer development and the poor outcomes.

### IL-37 suppresses colon cancer

**Figure 2:**
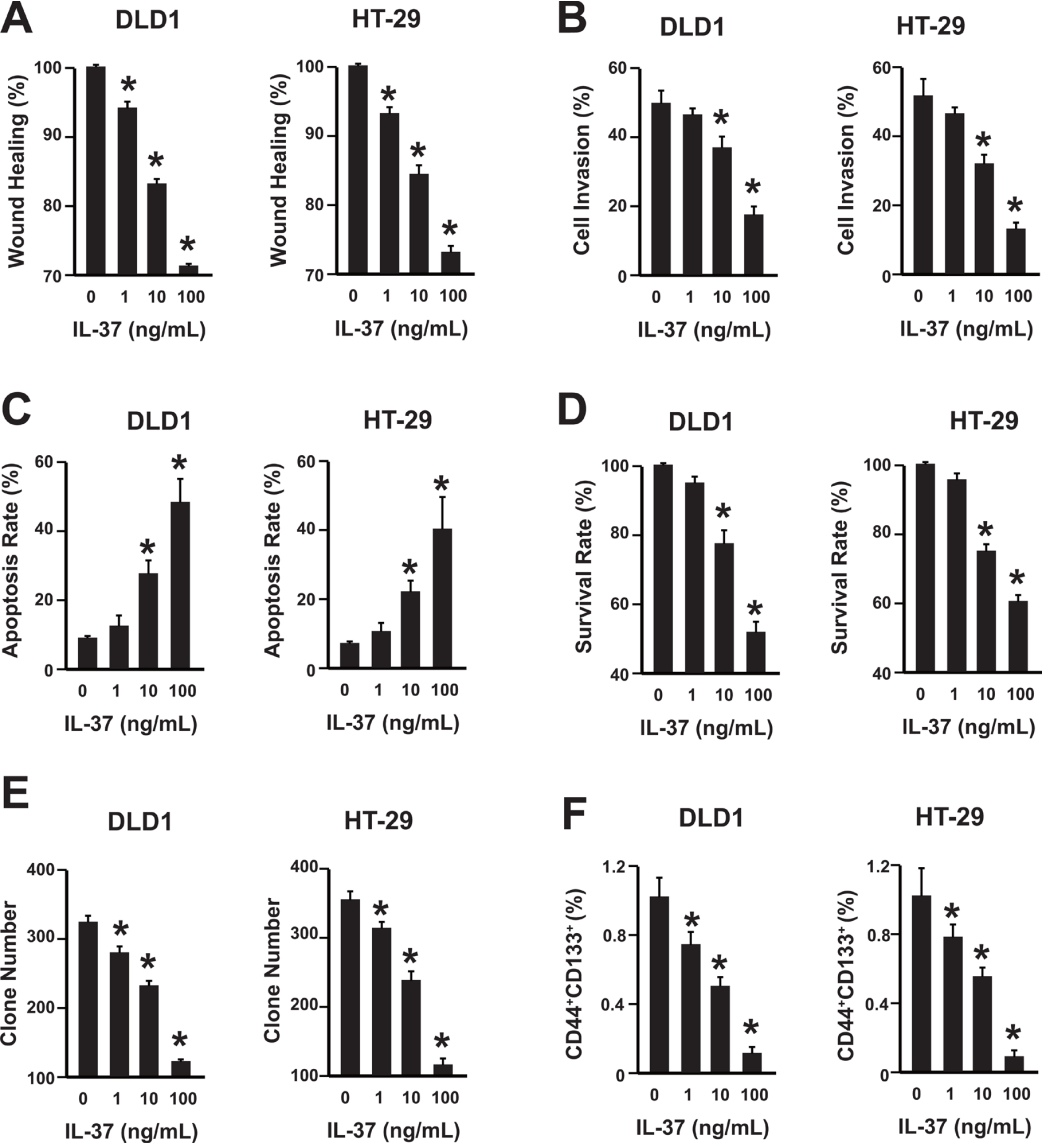
IL-37 suppresses colon cancer in a dose-dependent manner (**A**) Wound healing assay of DLD1 and HT-29 cells with different concentrations of rhIL-37 protein (0, 1, 10, 100 ng/mL). *n* = 3. *P < 0.05. (**B**) Cell invasion assay of DLD1 and HT-29 cells with different concentrations of rhIL-37 protein (0, 1, 10, 100 ng/mL). *n* = 3. *P < 0.05. (**C**) Analysis of colon cancer cell apoptosis following treatment of rhIL-37. DLD1 and HT-29 cells were treated at the indicated doses, harvested, and stained with Annexin V-FITC and 7-AAD. Annexin V-FITC-positive apoptotic cells were determined by flow cytometry. *n* = 3. *P < 0.05. (**D**) The survival rate of DLD1 and HT-29 cells treated with different concentrations of rhIL-37 (0, 1, 10, 100 ng/mL) were analyzed. *n* = 3. *P < 0.05. (**E**) The clone formation number of DLD1 and HT-29 cells treated with different concentrations of rhIL-37 (0, 1, 10, 100 ng/mL) were analyzed. *n* = 3. *P < 0.05. (**F**) The percentage of CD44^+^CD133^+^ cancer stem cells of DLD1 and HT-29 cells treated with different concentrations of rhIL-37 (0, 1, 10, 100 ng/mL) were analyzed. *n* = 3. *P < 0.05.

To uncover the mechanism of IL-37 in colon cancer development, cell proliferation, migration, invasion, and apoptosis were analyzed in human colon cancer cell line DLD1 and HT-29. The rhIL-37 was validated by western blot and its suppression effects on pro-inflammatory factors expression was confirmed by qPCR (Supplementary Figure 2). rhIL-37 suppressed the migration and invasion of DLD1 and HT-29 cells (Figure 2A, 2B). Additionally, rhIL-37 increased the apoptosis of DLD1 and HT-29 cells (Figure 2C). Moreover, rhIL-37 reduced the cell proliferation of DLD1 and HT-29 cells (Figure 2D). Furthermore, their clone formation capability and the percentage of colon cancer stem cell (CD44^+^CD133^+^ population) within colon cancer cells were also reduced by rhIL-37 (Figure 2E, 2F) [16–18].

Thus, rhIL-37 could suppress the cell migration, invasion, proliferation, and increase the colon cancer cell apoptosis. Furthermore, the rhIL-37 could reduce the number of cancer stem cells.

### IL-37 inhibits β-catenin expression in colon cancer cells

**Figure 3:**
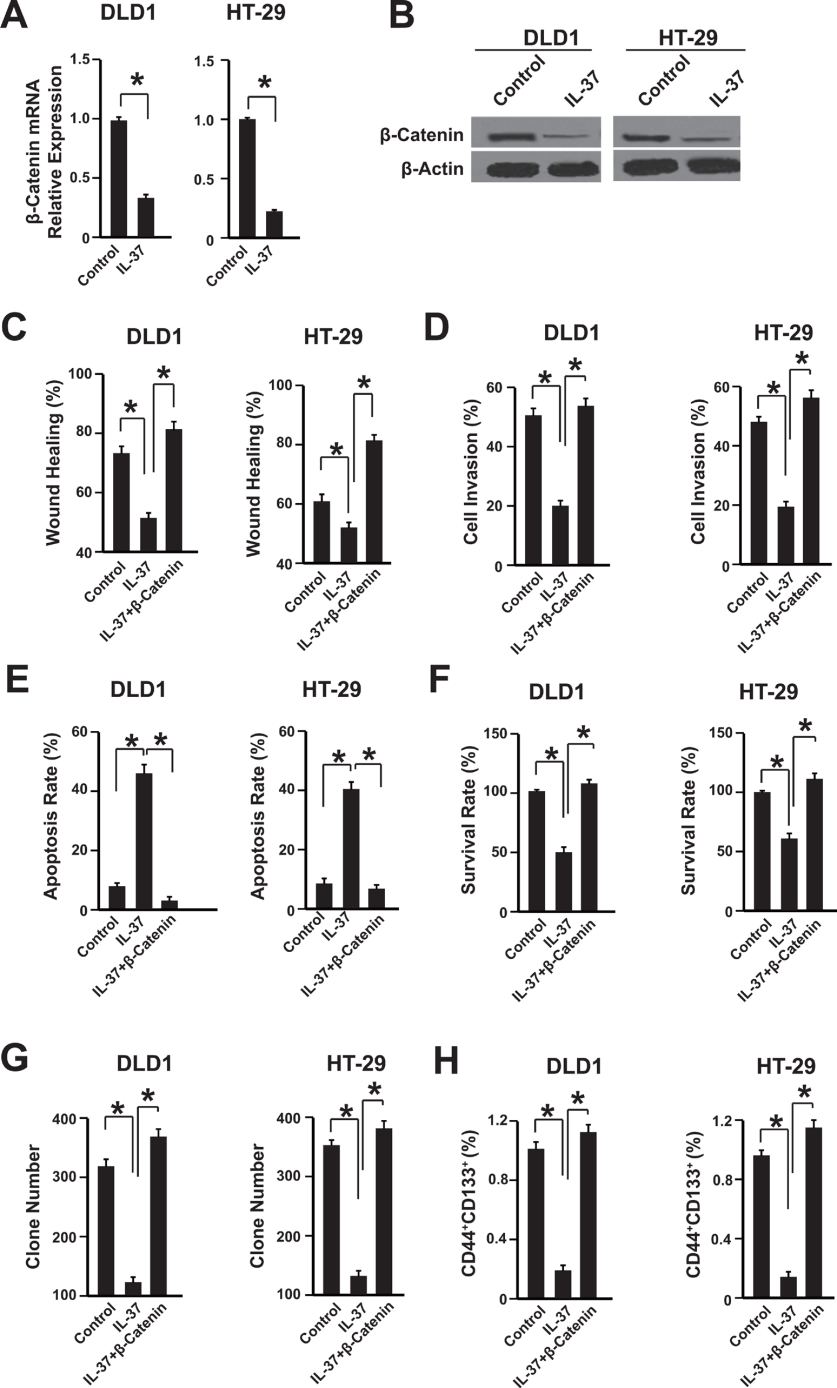
IL-37 inhibits β-catenin expression in colon cancer cells (**A**) The mRNA level of β-catenin was measured by qPCR. DLD1 and HT-29 cells were treated with 100 ng/mL rhIL-37. *n* = 3. *P < 0.05. (**B**) The protein level of β-catenin was measured by western blot. DLD1 and HT-29 cells were treated with 100 ng/mL rhIL-37. *n* = 3. *P < 0.05. (**C**–**H**) The wound healing, cell invasion, apoptosis, survival rate, clone formation capability and the percentage of cancer stem cells of cells overexpressing IL-37, β-catenin, both IL-37 and β-catenin or empty vector were analyzed. *n* = 3. *P < 0.05.

Because of the crucial role of β-catenin in colon cancer progression, the expression of β-catenin in DLD1 and HT-29 cells were analyzed [19]. Both mRNA and protein levels of β-catenin were suppressed by rhIL-37 (Figure 3A, 3B). Furthermore, the nuclear translocation of β-catenin was also reduced by IL-37 (Supplementary Figure 3). To further confirm the correlation between IL-37 and β-catenin signaling pathway, we transfected the DLD1 and HT-29 cells with plasmids overexpressing IL-37, β-catenin or them together. Compared with the control group, the effects of IL-37 on cell proliferation, migration, invasion, apoptosis and cancer stem cells were abolished in β-catenin overexpressing cells (Figure 3C–3H). These data demonstrated that IL-37 might inhibit colon cancer cells via β-catenin pathway.

### IL-37 overexpression inhibits colon cancer development *in vivo* and increases the sensitivity to chemotherapeutic drugs

**Figure 4:**
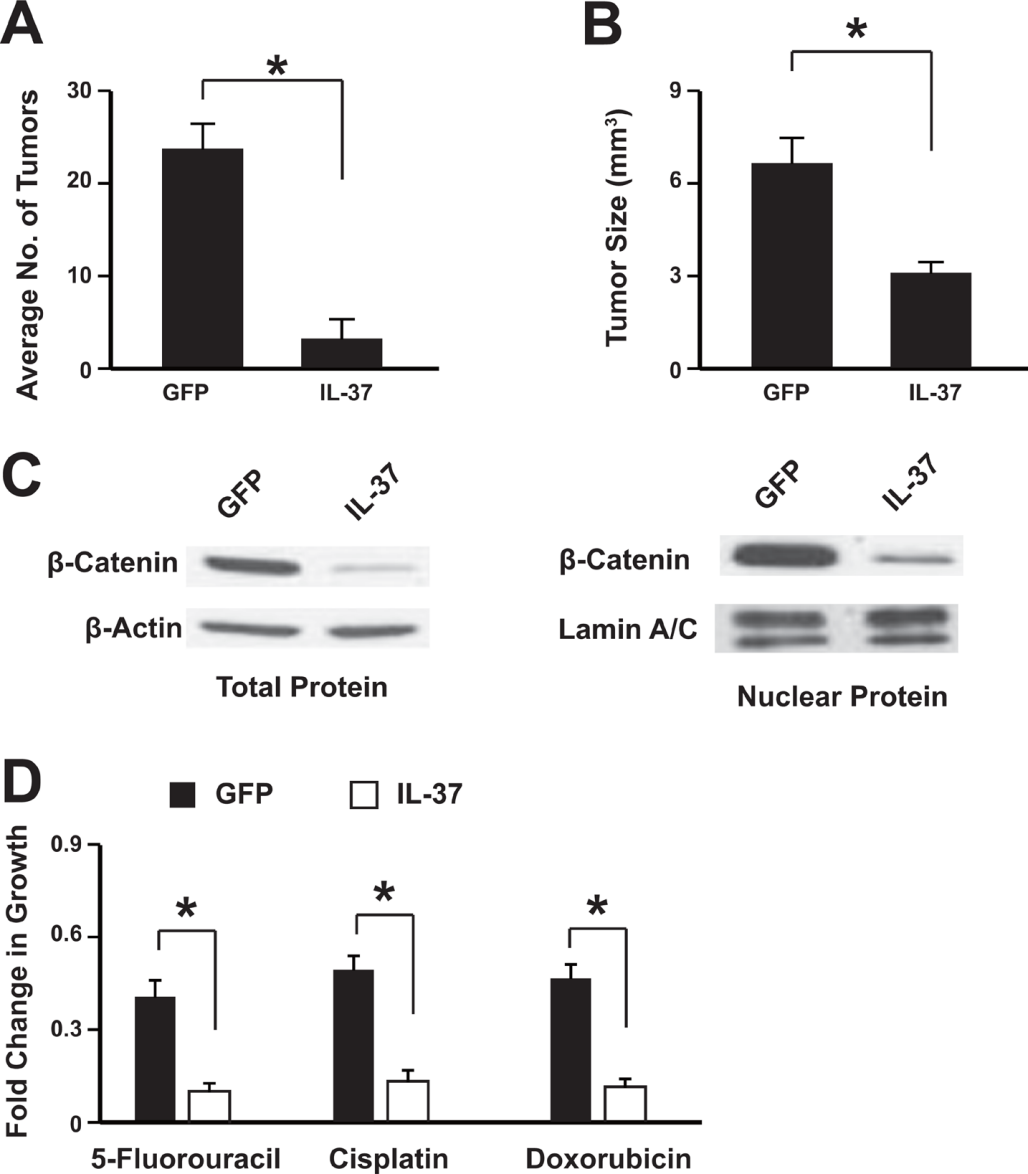
IL-37 suppresses colon tumorigenesis *in vivo* The adeno-associated virus expressing IL-37 or GFP was administrated via tail-vein injection. The average tumor number per mice (**A**) and the tumor size (**B**) were analyzed. The nuclear translocation of β-catenin in the tumors was assessed by western blot (**C**). Tumor cells were isolated from the mice and then subjected to chemotherapeutic drugs treatment (**D**). *n* = 12. *P < 0.05.

To study the anti-tumor effects of IL-37 *in vivo*, the colonic tumors were induced in mice with azoxymethane and DSS treatment. Then the adeno-associated virus expressing IL-37 or GFP was administrated via tail-vein injection. The results showed that mice treated with IL-37 had significantly less and smaller tumors than control mice (Figure 4A, 4B). Tumor cells were isolated from the mice and then subjected to further analysis. These isolated tumors were highly expressed IL-37 or GFP (Supplementary Figure 4A) and IL-37 suppressed the expression of inflammation factors (Supplementary Figure 4B, 4C). The β-catenin expression and its nuclear level were also suppressed in which expressed IL-37 (Figure 4C) . Chemotherapeutic drugs treatment showed that IL-37 sensitize the colon cancer to these drugs, including 5-Fluorouracil, Cisplatin and Doxorubicin (Figure 4D). These data showed that IL-37 might inhibit colon cancer development *in vivo* and sensitize the tumors to chemotherapy.

## DISCUSSION

Despite the current treatments for colon cancer having improved the survival significantly, the development of drug resistance still occurs in a great number of patients determining recurrence. Early diagnosis of colon cancer can improve survival rate, however, most patients are diagnosed at an advanced stage [1, 2]. Therefore, more efforts should be made to uncover the underlying mechanisms and develop novel therapeutic targets.

IL-37 is an anti-inflammatory and immune suppression factor [6]. Recent studies demonstrated that IL-37 has anti-tumor effects in mouse fibrosarcoma, human hepatocellular carcinoma and cervical cancer cells. In the mice model, overexpressing IL-37 suppressed fibrosarcoma, non-small cell lung cancer or hepatocellular carcinoma progression [9–12]. And the transgenic mice with IL-37 overexpression showed more resistance to DSS induced colitis [20]. But the underlying mechanisms of IL-37 in colon cancer remain unclear [12, 13]. Inflammation is one of the characteristics of cancer [21], indicating that IL-37 might inhibit cancer development through inflammation pathway suppression. Here, we provided the evidences that IL-37 inhibits the colon cancer progression via β-catenin suppression.

In the present study, data showed that the IL-37 was highly expressed in tumor paired normal tissues and the expression was decreased in cancer tissues. Further analysis showed that the lack of IL-37 tightly associated with cancer metastasis and poor survival, and could be developed as novel prognostic marker to predict tumor recurrence.

Then the colon cancer cell line DLD1 and HT-29 were treated with different concentration of IL-37. We found that IL-37 suppressed migration, invasion and proliferation of colon cancer cells. In the meantime, IL-37 also promoted colon cancer cell apoptosis and reduced cancer stem cell number.

Aberrantly activated β-catenin pathway results in colon cancer development [19]. Therefore, the β-catenin expression was assessed. We found that IL-37 down-regulated the expression of β-catenin at both mRNA and protein level. Furthermore, we also confirmed the anti-tumor effects of IL-37 in the mice model of colon cancer and demonstrated that IL-37 could sensitize the colon cancer cells to chemotherapeutic drugs.

The anti-tumor effects of IL-37 in colon cancer development might be very complicated. IL-37 inhibits the immune response and inflammation related factors. Therefore, inside of the tumor cells, IL-37 might suppress some factors which contribute to tumor development, such IL-6 or β-catenin we discussed here. But in the peri-tumor tissues, IL-37 would suppress the immune system. Thus, the high IL-37 level in cancer adjacent tissues might have the immune suppression effects and the low expression in the tumor tissue might have the tumor promotion effects. Both of them could promote tumor progression.

In conclusion, the data showed that colon cancer cells expressed very low level of IL-37. By inhibiting β-catenin expression, IL-37 impeded the colon cancer development. Therefore, IL-37 might be a potential target for colon cancer treatment. However, the mice homologue of IL-37 has not been found yet and the underlying mechanisms remain unclear. Thus, the mechanisms of anti-tumor effects of IL-37 need further studies.

## MATERIALS AND METHODS

### Patients

This was a retrospective study. A total of 186 pairs of samples were obtained from patients with primary colon cancer who had undergone surgery without any preoperative therapy at Liaoning Cancer Hospital and Institute, Cancer Hospital of China Medical University between January 2005 and December 2009. Patients with secondary colon cancer or already under preoperative therapies were excluded. Samples were collected from the patients consecutively with the standardized protocol. Every patient specimen included two matched pairs, namely, colon cancer tissues and adjacent normal colon tissues (≥ 5 cm away from the tumor). Surgically resected specimens were collected immediately after tumor removal and divided into two aliquots: half were immediately flash-frozen in liquid nitrogen and then frozen at −80°C until RNA and protein extraction was performed; the remainder was fixed with formalin for TMA (Tissue Microarrays) construction. The diagnosis was confirmed by at least two pathologists. The anatomical location of the primary tumor was categorized as right, transverse, left, or sigmoid. Staging was based on pathological findings according to the American Joint Committee on Cancer (AJCC). The study was conducted according to the Declaration of Helsinki and approved by the Ethics Committee of Cancer Hospital of China Medical University. Written informed consent was obtained from all patients.

### RNA extraction and real-time polymerase chain reaction (RT-PCR)

Total RNA was extracted from cells with Trizol (Invitrogen, Carlsbad, CA, USA) according to the manufacturer's instructions. Then the quantity and purity of RNA was determined by absorbance on a FilterMax F5 Multi-Mode Microplate Reader (Sunnyvale, California, USA) at 260 nm and 280 nm. Samples with ratios from 1.8 to 2.0 were accepted for next reverse transcription reaction. cDNA was prepared by using the iScript™ cDNA Synthesis kit (Bio-Rad, USA). PCR primers (Generay, Shanghai, China) used for RT-PCR were as follows: for IL-37, sense: 5′-AGTGCTGCTTAGAAGAC CCGG-3′ and anti-sense: 5′-AGAGTCCAGGACCAGTACTTTGTGA-3′; β-actin, sense: 5′-CCTGACTGACTACCTCATGAAG-3′ and anti-sense: 5′-GACGTAGCACAGCTTCTCCTTA-3′. RT-PCR amplification reaction was prepared with the SYBR Green PCR kit (Bio-rad, USA) and performed using the 7500 fast Real-Time PCR system (Applied Biosystems, USA). PCR products were verified by melting curve analysis. Relative mRNA levels of target genes were calculated by the 2^−ΔΔct^ method.

### Western blotting

Total protein from tumor tissues and cultured cells were lysed in RIPA buffer with protease inhibitor (Beyotime, Shanghai, China). The protein was quantified using a BCA assay kit (Beyotime, Shanghai, China). A total of 20 μg of total protein were separated by 10% SDS-PAGE, transferred onto polyvinylidene fluoride membranes, and then reacted with primary antibodies against IL-37, β-catenin and β-actin (all from Abcam, Cambridge, UK). After being extensively washed with PBS containing 0.1% Triton X-100, the membranes were incubated with alkaline phosphatase-conjugated goat anti-rabbit antibody for 30 min at room temperature. The bands were visualized using 1-step TM NBT/BCIP reagents (Thermo Fisher Scientific, Rockford, IL, USA) and detected by an Alpha Imager (Alpha Innotech, San Leandro, CA).

### Enzyme-linked immunoassay (ELISA)

The protein level of IL-37 was detected in culture supernatants and tumor homogenate using human IL-37 ELISA kit (AdipoGen AG, Liestal, Switzerland) according to the manufacturer's instructions. All samples were assayed in triplicate.

### TMA (Tissue Microarrays) construction and immunohistochemistry

Formalin-fixed, paraffin-embedded samples, including primary tumors and paired normal mucosa, were analyzed. Representative areas of tissue were established by microscopic review of H&E stained slides and 2.0mm diameter cores were punched from the paraffin blocks. Two cores from each sample at a distance of at least 2 cm from each other were arrayed. TMAs were created using a Tissue Microarrayer (Beecher Instruments, Sun Prairie, WI, USA). All specimens were examined by at least two pathologists to prevent bias. Tumor and normal mucosa morphology on the arrays were validated as having high accordance with that of the whole archived section by comparing the TMA staining and conventional HE staining of all tumor and paired normal tissues.

For IL-37 immunostaining, a microwave-based antigen retrieval process was employed with EDTA buffer, pH8.0, for 30 min. After the sections had been cooled, endogenous peroxidase was inhibited with 3% hydrogen peroxide for 10 min at room temperature. Non-specific binding was blocked with fetal calf serum for 15 min before incubation of the sections with mouse anti-human IL-37 antibody (ab57187, 1:1000, Abcam, Cambridge, MA, USA) at 4°C overnight. As a negative control, sections were incubated with normal mouse IgG. After being incubated with the primary antibodies, the sections were then incubated with horseradish peroxidase (HRP)-labeled anti-mouse IgG at 37°C for 30 min, followed by visualization with 3, 3-diaminobenzidine (DAB) and counterstaining with Mayer's hematoxylin. Desired color reaction was observed when monitored with the microscope.

Based on the intensity and extent of staining the immunohistochemically stained slides were reevaluated by two independent observers who were blinded to patient information. Briefly, IL-37 staining of the tumor cells was designated with an intensity score (0 [no staining], 1 [weak staining], 2 [moderate staining], and 3 [strong staining]) and an extent score (0 [no staining of cells], 1 [< 10% of tissue stained positive], 2 [10%–50% stained positive], 3 [> 50% stained positive]). The intensity and extent score were then summed up to give a total score ranging from 0 to 6, with a total score of 0 to 2, 3 to 4, and 5 to 6 defined as no expression, weak expression, and strong expression of IL-37.

### Recombinant human IL-37 protein expression

Interleukin-37 gene (homo species, isoform 1) was amplified from cDNA of peripheral blood mononuclear cell using the primer pair 5′-CGGGATCCATGGTTCACACAAGTCCA-3′ and 5′-CCCAAGCTTCTAATCGCTGACCTCACT-3′. The PCR fragments were double digested with restriction endonucleases and ligated into the prokaryotic expression vector. The fusion protein was expressed in a stable prokaryotic expression system. The plasmids of positive clones were then sequenced by Sanger method with 100% identify with the published sequence (GenBank: AF167368). The induced and un-induced cultures were analyzed by SDS-PAGE to identify the expression of recombinant protein. The harvested cells were resuspended in NaCl-Tris-HCl buffer, sonicated in an ice bath, 12000 rpm centrifuged for 30 min, and then the supernatant were collected. The supernatant were added to His Trap HP, 1 ml column (GE) that had been equilibrated with NaCl-Tris-HCl buffer. Different concentrations of imidazole buffer were used to elute the recombinant protein. Collected target protein peaks were examined by SDS-PAGE electrophoresis and immunoblot analysis using anti-human IL-37 antibody (Abcam, UK). The eluted recombinant protein was dialyzed in PBS at 4°C for overnight. The concentration was detected by Brandford methods, and the recombinant protein was stored at −20°C.

### Cell culture

The DLD1 and HT-29 human colon cell lines were obtained from the American Type Culture Collection (ATCC; Rockville, MD, USA) and cultured in DMEM (GIBCO, Shanghai, China) supplemented with 10% FBS. Recombinant human IL-37 (rhIL-37) protein, with a concentration ranging from 0 to 100 ng/mL (0, 1, 10, 100 ng/mL), was added to the medium of DLD1 and HT-29 cells after cultured for 24 h.

### Cell viability assay

Cell viability was evaluated using CCK-8 (Beyotime, Shanghai, China) according to manufacturer's instructions. Briefly, cells were seeded into 96-well plates at 5 × 10^3^ cells per well and cultured for indicated time points. 10 μl of CCK-8 solution was added into the culture medium in each well. After 1 hour incubation, OD values were read using a microplate reader (Bio-Tek Company, Winooski, VT, USA) at the 450-nm wavelength. Each time point was repeated in three wells and the experiment was independently performed for three times.

### Cell apoptosis assay

Cell apoptosis was evaluated by flow cytometry using an Annexin V-FITC Apoptosis Detection Kit (KeyGen Biotech Co. Roche, Nanjing, China). Briefly, cells were seeded into 24-well plates at 1 × 10^5^ cells per well and cultured for 48 h. Then the cells were detached by trypsinization, washed twice in PBS (2000 rpm, 5 min; Allegra X-12R centrifuge; Beckman Coulter, USA), and resuspended in 500 μL binding buffer. A volume of 5 μL Annexin V-FITC and 5 μL propidium iodide was added and mixed gently, and the cells were stained in the dark for 10 min at room temperature. The cells were analyzed immediately by flow cytometry (BD FACSCalibur, BD Bioscience, San Diego, CA, USA) and analyzed using Flowjo software (FlowJo, Ashland, OR, USA). The experiment was repeated three times.

### Cell migration assay

The migration of cells was detected by wound-healing assay. Cells were cultured in 6-well plates. When the cells grew to 80–90% confluence, a wound in a line across the well was made by a plastic pipette tip. The area of cell-free wound was recorded 24 h after incubation with rhIL-37 protein using an inverted microscope and analyzed by the NIH Image 1.55 software.% Wound healing = 100x (1-the remaining cell-free area/the area of the initial wound). All tests were performed in triplicate.

### Transwell invasion assay

Invasive ability of cells was determined within a transwell system. 6.0 ×10^4^ cells were seeded onto the upper surface of the transwell membrane and cultured at 37°C in 5% CO_2_ for 24 h, 48 h and 72 h. The number of cells that migrated to the lower surface of the membrane was counted under a microscope (200×).

### Clonogenic assays

The cells were plated at 1 × 10^3^ cells per p100 plate in standard growth media. The cells were allowed to form colonies for 10 days before being fixed and stained with 0.2% crystal violet (w:v) in 10% buffered formalin. Colony numbers were manually counted.

### Flow cytometry

Two million cells were harvested from 85% confluent flasks and resuspended in PBS with 0.1% BSA. The cells were washed and incubated at 4°C for 30 minutes with anti-CD44-allophycocyanin (APC) (1:20 dilution, clone G44-26, BD Biosciences) and anti-CD133-phycoerythrin (PE) (1:20 dilution, clone AC133, MiltenyiBiotec) antibodies, or mouse-specifc IgG2b ĸ-APC (1:100dilution, BD Biosciences) and IgG1-PE (1:20 dilution, Miltnyi Biotec) antibodies. The cells were then washed and resuspended in PBS with 0.1% BSA and 2 μg/mL propidium iodide (PI), and a C6 FACS (BD Biosciences) was used for all analyses. The cells were first gated on the basis of side-scatter and forward-scatter, followed by the exclusion of nonviable (PI-positive) cells. The CD44^+^ and CD133^+^ gates were created on the basis of cellular staining with the isotype control antibodies (IgG2bĸ-APC and IgG1-PE, respectively).

### Animal study

6–8 weeks old C57 mice (Charles River Laboratories, Beijing, China) were housed in specific pathogen-free conditions. The mice used in current study are one inbred strain of C57BL/6J which had been exposed to various stimulations before. This strain is more susceptible to colon cancer development under AOM/DSS stimulation (data not published and under preparation for publication). The study was approved by the Research Ethics Committee of Cancer Hospital of China Medical University. Mice were housed in the pathogen free region and monitored daily during the experiments and the mice would be sacrificed when the weight loss is more than 20%. Mice were anesthetised with an intraperitoneal injection of ketamine 100 mg/kg and xylazine 10 mg/kg.

For induction of colonic tumors, mice were first administered an intraperitoneal injection of azoxymethane (7.4mg/kg, Sigma-Aldrich, USA); one week later, 1% DSS was first administered for 7 days in drinking water, then followed by drinking distilled water for 3 weeks. Finally the mice were killed. All polypoid or flat elevated lesions that developed were histo-pathologically counted by observation of a longitudinal paraffin section with H&E staining. Tumor size was measured with fine digital calipers and calculated by the following formula: tumor volume =0.5 × width^2^ × length.

### Production and *in vivo* delivery of Adeno-associated Virus

Vector construction, production, and *in vivo* delivery of adeno-associated virus (AAV) were performed based on the AAV helper-free system (Agilent). The recombinant adenoviral vector pAAV-IL37 was constructed by cloning the cDNA encoding region into pAAV-ITR. The vector pAAV-GFP encoding green fluorescence protein was used as a negative control. Recombinant AAVs were produced by HEK293 cells (ATCC) transfected with pAAV-ITR vectors together with pAAV-RC and pHelper plasmids, and then purified by discontinuous iodixanol gradient centrifugation. Purified recombinant AAVs were concentrated and desalted by centrifugation through Amicon Ultra 30K filters (Millipore). For *in vivo* delivery, recombinant AAVs equivalent to 1.0 × 10^12^ viral genome copies were delivered though mouse tail vein.

### Chemotherapy assay

For cell growth in the presence of chemotherapeutic agents, cells were seeded at 5 × 10^4^ cells per well and treated the following day with 5-Fluorouracil (1.5 μM, Sigma), Cisplatin (1.5 μM, Sigma), and Doxorubicin (30 nM, Sigma) in standard growth medium for 24 hours. Following treatment, cells were plated in triplicate at 1 × 10^3^ in 6-well plates. On day 10, cells were stained with crystal violet, solubilized with 1% SDS and measured for absorbance at 590 nm.

### Statistical analysis

Data were expressed as mean (± SE) and analyzed by a SPSS software package (SPSS Standard version 13.0, SPSS Inc, USA). Differences between variables were assessed by the Chi-square test. Survival analysis of patients with colorectal cancer was calculated by Kaplan-Meier analysis. A log rank test was used to compare different survival curves. A Cox proportional hazards model was used to calculate univariate and multivariate hazard ratios for the variables. Unpaired Student's t test and one way ANOVA were used as appropriate to assess the statistical significant of difference. *P* values under 0.05 were considered statistically significant.

## CONCLUSIONS

IL-37 is decreased in human colon cancer and capable of exerting anti-tumor activity by suppressing the β- catenin expression. It might be developed as a promising prognostic predictor and therapeutic target in the treatment of colon cancer.

## SUPPLEMENTARY FIGURES



## References

[1] Siegel RL, Miller KD, Jemal A (2016). Cancer statistics, 2016. CA Cancer J Clin.

[2] Siegel R, Desantis C, Jemal A (2014). Colorectal cancer statistics, 2014. CA Cancer J Clin.

[3] Worthley DL, Whitehall VL, Spring KJ, Leggett BA (2007). Colorectal carcinogenesis: road maps to cancer. World J Gastroenterol.

[4] Pancione M, Giordano G, Remo A, Febbraro A, Sabatino L, Manfrin E, Ceccarelli M, Colantuoni V (2014). Immune escape mechanisms in colorectal cancer pathogenesis and liver metastasis. J Immunol Res.

[5] Zhong Z, Sanchez-Lopez E, Karin M (2016). Autophagy, Inflammation, and Immunity: A Troika Governing Cancer and Its Treatment. Cell.

[6] Nold MF, Nold-Petry CA, Zepp JA, Palmer BE, Bufler P, Dinarello CA (2010). IL-37 is a fundamental inhibitor of innate immunity. Nat Immunol.

[7] Chen HM, Fujita M (2015). IL-37: a new player in immune tolerance. Cytokine.

[8] Banchereau J, Pascual V, O'Garra A (2012). From IL-2 to IL-37: the expanding spectrum of anti-inflammatory cytokines. Nat Immunol.

[9] Wang S, An W, Yao Y, Chen R, Zheng X, Yang W, Zhao Y, Hu X, Jiang E, Bie Y, Chen Z, Ouyang P, Zhang H, Xiong H (2015). Interleukin 37 Expression Inhibits STAT3 to Suppress the Proliferation and Invasion of Human Cervical Cancer Cells. J Cancer.

[10] Gao W, Kumar S, Lotze MT, Hanning C, Robbins PD, Gambotto A (2003). Innate immunity mediated by the cytokine IL-1 homologue 4 (IL-1H4/IL-1F7) induces IL-12-dependent adaptive and profound antitumor immunity. J Immunol.

[11] Zhao JJ, Pan QZ, Pan K, Weng DS, Wang QJ, Li JJ, Lv L, Wang DD, Zheng HX, Jiang SS, Zhang XF, Xia JC (2014). Interleukin-37 mediates the antitumor activity in hepatocellular carcinoma: role for CD57+ NK cells. Sci Rep.

[12] Ge G, Wang A, Yang J, Chen Y, Yang J, Li Y, Xue Y (2016). Interleukin-37 suppresses tumor growth through inhibition of angiogenesis in non-small cell lung cancer. J Exp Clin Cancer Res.

[13] Chen YH, Zhou BY, Wu XJ, Xu JF, Zhang JA, Chen YH, Liang SS (2016). CCL22 and IL-37 inhibit the proliferation and epithelial-mesenchymal transition process of NSCLC A549 cells. Oncol Rep.

[14] Jiang Y, Wang Y, Liang L, Gao Y, Chen J, Sun Y, Cheng Y, Xu Y (2015). IL-37 mediates the antitumor activity in renal cell carcinoma. Med Oncol.

[15] Wang WQ, Zhao D, Zhou YS, Hu XY, Sun ZN, Yu G, Wu WT, Chen S, Kuang JL, Xu GG, Han ZC, Wang BM, Yang JX, Feng XM (2015). Transfer of the IL-37b gene elicits anti-tumor responses in mice bearing 4T1 breast cancer. Acta Pharmacol Sin.

[16] Haraguchi N, Ohkuma M, Sakashita H, Matsuzaki S, Tanaka F, Mimori K, Kamohara Y, Inoue H, Mori M (2008). CD133+CD44+ population efficiently enriches colon cancer initiating cells. Ann Surg Oncol.

[17] Galizia G, Gemei M, Del Vecchio L, Zamboli A, Di Noto R, Mirabelli P, Salvatore F, Castellano P, Orditura M, De Vita F, Pinto M, Pignatelli C, Lieto E (2012). Combined CD133/CD44 expression as a prognostic indicator of disease-free survival in patients with colorectal cancer. Arch Surg.

[18] Bellizzi A, Sebastian S, Ceglia P, Centonze M, Divella R, Manzillo EF, Azzariti A, Silvestris N, Montemurro S, Caliandro C, De Luca R, Cicero G, Rizzo S (2013). Co-expression of CD133(+)/CD44(+) in human colon cancer and liver metastasis. J Cell Physiol.

[19] Morin PJ, Sparks AB, Korinek V, Barker N, Clevers H, Vogelstein B, Kinzler KW (1997). Activation of beta-catenin-Tcf signaling in colon cancer by mutations in beta-catenin or APC. Science.

[20] McNamee EN, Masterson JC, Jedlicka P, McManus M, Grenz A, Collins CB, Nold MF, Nold-Petry C, Bufler P, Dinarello CA, Rivera-Nieves J (2011). Interleukin 37 expression protects mice from colitis. Proc Natl Acad Sci U S A.

[21] Hanahan D, Weinberg RA (2011). Hallmarks of cancer: the next generation. Cell.

