# Supplementary material for: Interleukin-37 mediates the antitumor activity in colon cancer through β-catenin suppression
Source: Oncotarget. 2017 Apr 13;8(30):49064–75. doi: 10.18632/oncotarget.17093 (PMC5564749; doi:10.18632/oncotarget.17093)
Supplement: Supplementary file 1 [file oncotarget-08-49064-s001.pdf]

Interleukin-37 mediates the antitumor activity in colon cancer through β-catenin suppression

Supplementary Materials

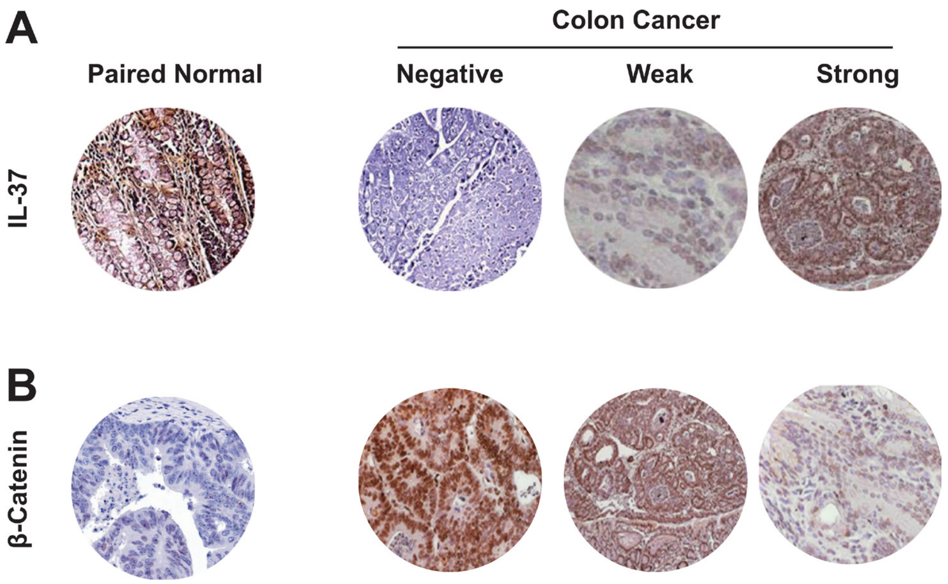

Supplementary Figure 1: IL-37 and β-catenin expression in human colon cancer biopsies and paired normal tissues. (**A**) Representative color photomicrographs negative, weak, and strong IL-37 expression. (**B**) Representative color photomicrographs of β-catenin expression in colon cancer biopsies with negative, weak, and strong IL-37 expression.

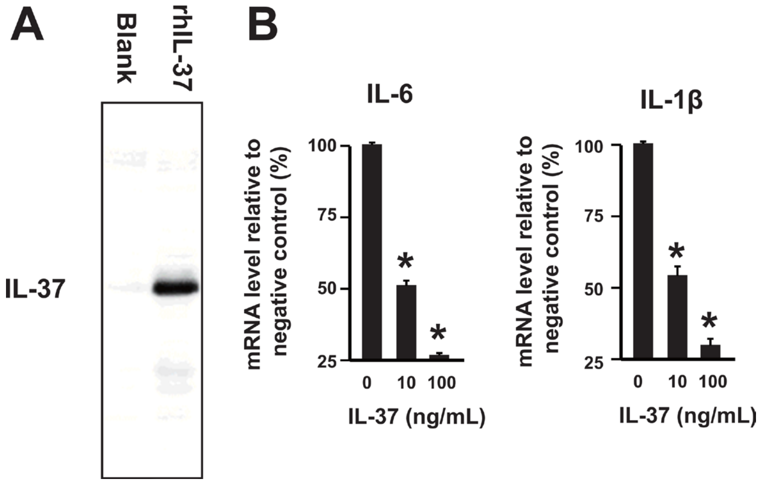

Supplementary Figure 2: IL-37 suppresses colon cancer in a dose-dependent manner. (**A**) SDS-PAGE and immunoblot for the recombinant human (rh) IL-37. (**B**) The mRNA levels of pro-inflammatory factor IL-6 and IL1-β were measured in peripheral blood mononuclear cells of patients with colon cancer when treated with different concentrations of rhIL-37 (0, 10, 100 ng/mL). $n = 3$. $^*P < 0.05$.

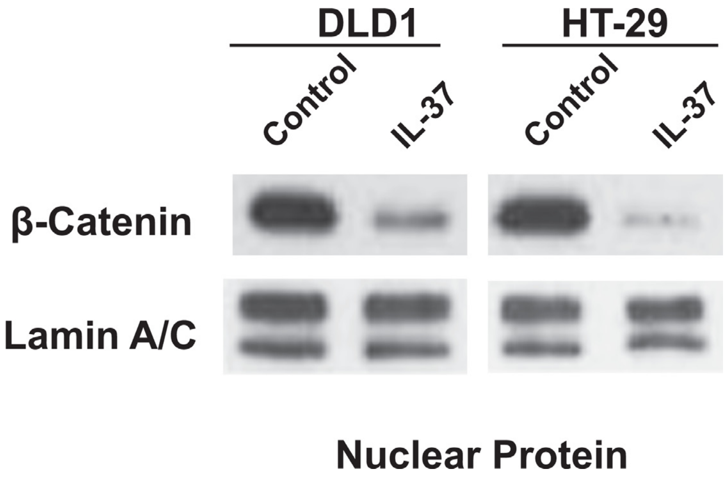

Supplementary Figure 3: The nuclear translocation of activated β-catenin was assessed in DLD1 and HT-29 cells by western blot. Nuclear protein was isolated with NE-PER™ Nuclear and Cytoplasmic Extraction Reagents (Thermo Scientific, Cat No. 78833) and Lamin A/C was used as nuclear protein control.

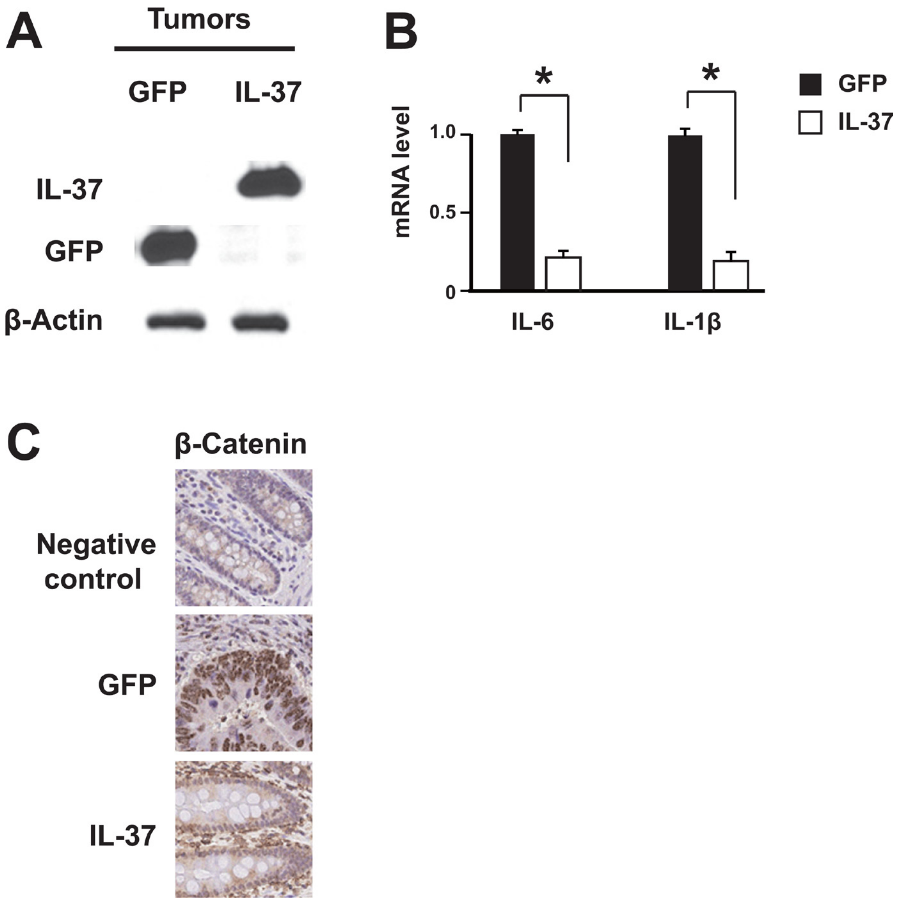

Supplementary Figure 4: (**A**) The GFP and IL-37 expression in the mice colon tumor was validated with western blot. Sample was collected at the end of the experiments. (**B**) The mRNA level of pro-inflammatory factor IL-6 and IL1-β were measured in mice colon cancer tissues by qPCR. (**C**) Representative histopathology figure for mice colon tumors tissues and the β-catenin expression was detected by immunostaining.
